# The complete chloroplast genome of *Cotoneaster schantungensis*

**DOI:** 10.1080/23802359.2019.1681923

**Published:** 2019-10-24

**Authors:** Xue-Yun Li, Wei Li, Qing-hua Liu, Xiao-Man Xie, Yi-Zeng Lu, Cui-Ping Zhang

**Affiliations:** a College of Landscape Architecture and Forestry, Qingdao Agricultural University, Qingdao, China;; b Shandong Provincial Center of Forest Tree Germplasm Resources, Jinan, China

**Keywords:** *Cotoneaster schantungensis*, chloroplast genome, phylogenetic analysis

## Abstract

*Cotoneaster schantungensis* is an endangered vulnerable species restricted to Shandong Province. In this study, we report the sequencing of the *C. schantungensis* chloroplast (cp) genome using the Illumina Novaseq platform for the first time. The complete *C. schantungensis* cp genome is 159,883 bp in length and contains two inverted repeats (IRs), separated by a large single-copy region (LSC) and a small single-copy region (SSC). A total of 132 unique coding genes, including 85 protein-coding genes, 39 tRNA genes, and 8 rRNA genes were identified. The maximum likelihood phylogenetic analysis revealed that *C. schantungensis* is closely related to *Eriobotrya japonica.*

*Cotoneaster schantungensis* is a perennial deciduous shrub in genus *Cotoneaster* of the Rosaceae family. It is only distributed in Shandong province and has its fruits and flowers possess excellent ornamental values. *C. schantungensis* has been classified as a critically endangered species. There are currently very few molecular studies on *C. schantungensis* and its genetic information is extremely scarce. Previous studies on *C. chantungensis* mainly focussed on transcriptome sequencing (Bu et al. 2019), primer screening (Bu et al. 2019), community structure determination (Qu et al. 2012), and propagation technique development (Li et al. 2014). Here, we characterize the cp genome of *C. chantungensis* using the Illumina Novaseq platform, aiming to provide valuable genetic information for future *C. chantungensis* research and germplasm conservation.

Fresh and healthy leaf tissues were collected from the Hongye Valley in Jinan, Shandong, China (36°28′N, 117°09′E). The voucher specimen (accession no. QAU20190521) was stored at –80 °C at the Qingdao Agricultural University. Genomic DNA was extracted using the CTAB method with minor modifications. A library with an average length of 350 bp was constructed using the NexteraXT DNA Library Preparation Kit (manufacture’s info). Approximately 5.77 Gb of raw paired-end reads of 150 bp were obtained and low-quality reads were removed, yielding 5.73 GB clean data that were subsequently assembled into contigs using SPAdes v3.10.1 (Anton et al. 2012). The contigs were then further assembled into the complete *C. schantungensis* cp genome using NOVOPlasty version 2.6.2 (Dierckxsens et al. 2017) and gene annotation was performed by CpGAVAS (Liu et al. 2012) and DOGMA (Boore et al. 2004). A circular cp genome map of *C. schantungensis* was constructed in OGDRAW. The annotated *C. schantungensis* cp genome sequence was deposited to NCBI under an accession no. MN457692.

The size of *C. schantungensis* cp genome is 159,883 bp, and similar to that of other higher plants, it displays a typical quadripartite structure, which contains two inverted repeats (each was 26,397 bp in length) separated by a large single copy region (LSC, 87,864 in length) and a small single-copy region (SSC, 19,225 bp in length). The GC content is 38.6% and we identified 132 unique genes, including 85 protein-coding genes, 39 tRNA genes, and 8 rRNA genes. Among the unique genes, 9 protein-coding genes and 6 tRNA genes contain only one intron, and two protein-coding genes, *ycf*3 and *clpP*, display two introns. In addition, trans-splicing was detected for the *rps12* gene.

Fourteen publicly available cp genomes were included in the phylogenetic analysis by MAFFT v7.307 (Katoh and Standley 2013). The phylogenetic tree constructed using the maximum likelihood (ML) method in RAxML8.0 (Stamatakis 2014) revealed a close relationship of *C. schantungensis* with *Eriobotrya japonica* compared with other taxa (Figure 1). This result is consistent with previous taxonomic studies. Our findings provide valuable genetic information of *C. schantungensis* for future genetic diversity studies and germplasm conservation.

**Figure 1.**
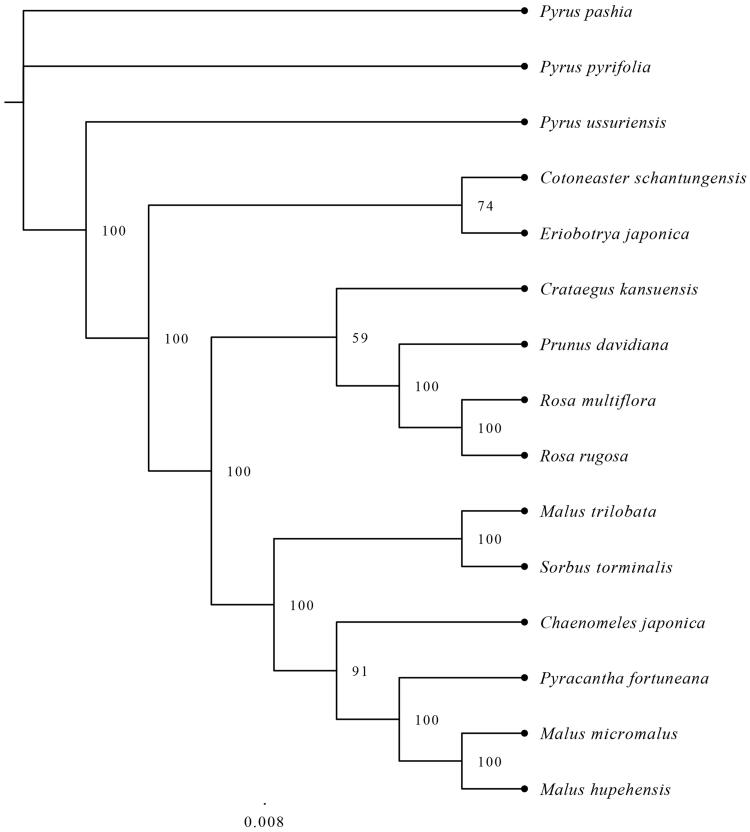
Phylogenetic relationships among 14 *Rosaceae* species based on their complete cp genomes.

Accession number: (*Sorbus torminalis*, NC 033975; *Pyrus ussuriensis*, NC 041461; *Pyrus pyrifolia*, NC 015996; *Pyrus pashia*, NC 034909; *Pyracantha fortuneana*, NC 042192; *Rosa multiflora*, NC 039989; *Rosa rugosa*, NC 044094; *Prunus davidiana*, NC 039735; *Malus trilobata*, NC 035671; *Malus micromalus*, NC 036368; *Malus hupehensis*, NC 040170; *Eriobotrya japonica*, NC 034639; *Chaenomeles japonica*, NC 035566; *Crataegus kansuensis*, NC 039374)

## References

[CIT0001] Anton B, Sergey N, Dmitry A, Gurevich AA, Mikhail D, Kulikov AS, Lesin VM, Nikolenko SI, Son P, Prjibelski AD 2012 SPAdes: a new genome assembly algorithm and its applications to single-cell sequencing. J Comput Biol. 19:455–477. 22506599 10.1089/cmb.2012.0021 PMC3342519

[CIT0002] Boore JL, Jansen RK, Wyman SK 2004 Automatic annotation of organellar genomes with DOGMA. Bioinformatics. 20:3252–3255. 15180927 10.1093/bioinformatics/bth352

[CIT0003] Bu JY, Ni DQ, Zang DK 2019 Full-length transcriptome sequencing assembly and gene functional annotation of *Cotoneaster schantungensis*. Mol Plant Breeding. 2:1–9.

[CIT0004] Bu JY, Zang DK 2019 Extraction of genomic DNA from *Cotoneaster schantungensis* and screening of SRAP molecular marker primers. J Anhui Agric Sci. 47:3.

[CIT0005] Dierckxsens N, Mardulyn P, Smits G 2017 NOVOPlasty: de novo assembly of organelle genomes from whole genome data. Nucleic Acids Res. 45:e18. 10.1093/nar/gkw955 PMC5389512 28204566

[CIT0006] Katoh K, Standley DM 2013 MAFFT multiple sequence alignment software version 7: improvements in performance and usability. Mol Biol Evol. 30:772–780. 23329690 10.1093/molbev/mst010 PMC3603318

[CIT0007] Li ZH, Li WQ, Xie XM, Ning H, Zang DK, Guo XF 2014 Study on cutting propagation of rare and endangered plant *Cotoneaster schantungensis*. Shandong Forest Sci Tech. 2:69–71.

[CIT0008] Liu C, Shi LC, Zhu YJ, Chen HM, Zhang JH, Lin XH, Guan XJ 2012 CpGAVAS, an integrated web server for the annotation, visualization, analysis, and GenBank submission of completely sequenced chloroplast genome sequences. BMC Genomics. 13:715. 23256920 10.1186/1471-2164-13-715 PMC3543216

[CIT0009] Qu SQ, Liu D, Xie XM, Lin QH, Zang DK 2012 Study on composition and structure characteristics of *Cotoneaster schantungensis* community. J Anhui Agri Sci. 40:1426–1427, 1430.

[CIT0010] Stamatakis A 2014 RAxML version 8: a tool for phylogenetic analysis and post-analysis of large phylogenies. Bioinformatics. 30:1312–1313. 24451623 10.1093/bioinformatics/btu033 PMC3998144

